# Mapping the risk of respiratory infections using suburban district areas in a large city in Colombia

**DOI:** 10.1186/s12889-023-16179-5

**Published:** 2023-07-20

**Authors:** Javier Cortes-Ramirez, Michelle Gatton, Juan D. Wilches-Vega, Helen J. Mayfield, Ning Wang, Olga M. Paris-Pineda, Peter D. Sly

**Affiliations:** 1 Centre for Data Science, Queensland University of Technology, Brisbane City, Australia; 2 Children’s Health and Environment Program, Child Health Research Centre, The University of Queensland, St Lucia, Australia; 3 Faculty of Health, University of Santander, Santander, Colombia; 4 Centre for Immunology and Infection Control, Queensland University of Technology, Brisbane City, Australia; 5 School of Public Health, The University of Queensland, St Lucia, Australia; 6 National Centre for Chronic and Noncommunicable Disease Control and Prevention. Chinese Centre for Disease Control and Prevention, Beijing, China; 7 Queensland University of Technology, O Block D Wing Room D722. Ring Road, Kelvin Grove Campus, Victoria Park Road. Kelvin Grove, Kelvin Grove, QLD 4059 Australia

**Keywords:** Acute respiratory infections, Bayesian spatial hierarchical regression model, Besag-York-Mollie specification -BYM, Integrated Nested Laplace approximation -INLA, Cúcuta / Cucuta –North Santander, Colombia, Suburban areas, Sociodemographic and environmental risk factors, Hotspots or risk, Spatial epidemiological analysis

## Abstract

**Background:**

Acute respiratory infections (ARI) in Cúcuta -Colombia, have a comparatively high burden of disease associated with high public health costs. However, little is known about the epidemiology of these diseases in the city and its distribution within suburban areas. This study addresses this gap by estimating and mapping the risk of ARI in Cúcuta and identifying the most relevant risk factors.

**Methods:**

A spatial epidemiological analysis was designed to investigate the association of sociodemographic and environmental risk factors with the rate of ambulatory consultations of ARI in urban sections of Cúcuta, 2018. The ARI rate was calculated using a method for spatial estimation of disease rates. A Bayesian spatial model was implemented using the Integrated Nested Laplace Approximation approach and the Besag-York-Mollié specification. The risk of ARI per urban section and the hotspots of higher risk were also estimated and mapped.

**Results:**

A higher risk of IRA was found in central, south, north and west areas of Cúcuta after adjusting for sociodemographic and environmental factors, and taking into consideration the spatial distribution of the city’s urban sections. An increase of one unit in the percentage of population younger than 15 years; the Index of Multidimensional Poverty and the rate of ARI in the migrant population was associated with a 1.08 (1.06—1.1); 1.04 (1.01—1.08) and 1.25 (1.22—1.27) increase of the ARI rate, respectively. Twenty-four urban sections were identified as hotspots of risk in central, south, north and west areas in Cucuta.

**Conclusion:**

Sociodemographic factors and their spatial patterns are determinants of acute respiratory infections in Cúcuta. Bayesian spatial hierarchical models can be used to estimate and map the risk of these infections in suburban areas of large cities in Colombia. The methods of this study can be used globally to identify suburban areas and or specific communities at risk to support the implementation of prevention strategies and decision-making in the public and private health sectors.

**Supplementary Information:**

The online version contains supplementary material available at 10.1186/s12889-023-16179-5.

## Introduction

Acute Respiratory Infections (ARI), including both upper and lower respiratory infections, are major causes of morbidity and mortality in all age groups, especially in low- and middle-income countries [1, 2]. Extensive research exists on the physiopathology of these diseases and their overall association with individual risk factors. These factors include environmental conditions such as indoor and outdoor air-quality which are often affected by burning biofuels [3], wildfires [4] and traffic congestion [5], and climatic factors such as temperature and precipitation [6, 7]. Social and demographic characteristics have also been relevant, including poverty [8, 9], immigration status [10], ethnicity [4, 9], and age for both the very young and the elderly being at higher risk [11]. Recent studies have further identified the role of geographic variability in the risk of ARI, and the need to understand the spatial distribution of risk at both the state [6, 12] and at finer census district levels [7, 10]. To use these findings in a practical public health decision making context, it is therefore important to understand how risk varies at a local level. The spatial heterogeneity of the infection risk can be considered in epidemiological analyses to increase the accuracy of prediction of outbreaks and identifying potential underlying factors determinant of these infections [13].

In Colombia, the morbidity of ARI represents 5% of all ambulatory consultations and 7% of all hospitalisations, with the North Santander region and its capital city Cúcuta having a comparatively higher burden of disease [14]. Despite the health impacts and public health costs associated with ARI, few studies have investigated the epidemiology and risk factors of these diseases in the region. Previous studies have used surveys to identify the association of poor air quality with a higher incidence of ARI in specific areas or targeted communities such as childcare centres in Cúcuta [15, 16]. However, there is little research on the association of these diseases with social, demographic and environmental risk factors and for larger areas or communities in the region. This gap can be explained in part because of the absence of high-quality morbidity data. The Department of Health provides statistics on ARI for each health centre and hospital in Cúcuta, but this does not include information such as specific disease groups and the patient’s residence. These data limitations can impact the effectiveness of decision-making in public health since analyses of basic statistics might not be sufficient to identify the most at-risk populations, and therefore exacerbate the role that external factors such as political trends have on shaping public health initiatives in place of an evidence-based approach [17].

An important limitation of the statistics on ARI in Cúcuta is that these data are not linked to key information such as the place of residence which prevents the calculation of indicators for specific areas. However, previous research has introduced a method to estimate the cases of ARI in Cúcuta for high-spatial-resolution areas aligned with the census districts, using data from the Department of Health [18]. In the context of limited health data, spatial analysis methods provide a robust tool for epidemiological studies to estimate and map disease risk. Spatial epidemiology is becoming a common approach to estimate the morbidity risk of infections in Colombia, especially for vector borne diseases such as Zika, dengue fever, malaria and chikungunya [19–23]. Although previous studies have mapped the risk of some infectious diseases in North Santander [24], spatial analyses on the risk of respiratory infections have not been implemented in Cúcuta or other cities in the region.

Spatial statistics can be used to analyse health data in suburban areas to identify significant determinants of diseases such as ARI. Spatial regression models incorporate the spatial structure of the data (i.e., their interrelation between geographical areas) to increase the robustness of risk estimates [25, 26]. Bayesian models are especially effective for analyses of spatially distributed data due to their suitability to specify hierarchical structures in the data that need to be taken into account for statistical inference [27]. The Bayesian approach has been increasingly used due to the higher computation capabilities developed in the last three decades and the extended use of these methods in epidemiological studies [28]. An additional advantage of Bayesian analyses is they allow the development of risk maps which is of particular interest to study the morbidity in suburban areas of large cities such as Cúcuta. The present study aims to map the risk of ARI in Cúcuta and to identify the most relevant risk factors for these diseases. The objectives are to estimate the association of socioenvironmental risk factors with ARI in Cúcuta, and to estimate the risk in specific suburban areas and identify hotspots of risk, using a hierarchical Bayesian spatial model. This analysis would allow the identification of specific communities at risk to support directed and more effective surveillance and prevention strategies.

## Methods

The study followed an ecological design, using census districts as the ecological units. A Bayesian spatial regression was used to estimate the association of sociodemographic and environmental risk factors with the incidence rate of ARI in Cúcuta, in the 12-month period from 1st January to 31st December 2018. The risk of ARI was then mapped, and a cluster analysis was implemented to identify the hotspots of higher risk. This study was approved by the ethics committee of the University of Santander (VII-FT-025-UDES, 021 25/06/2019).

### Study area

**Fig. 1:**
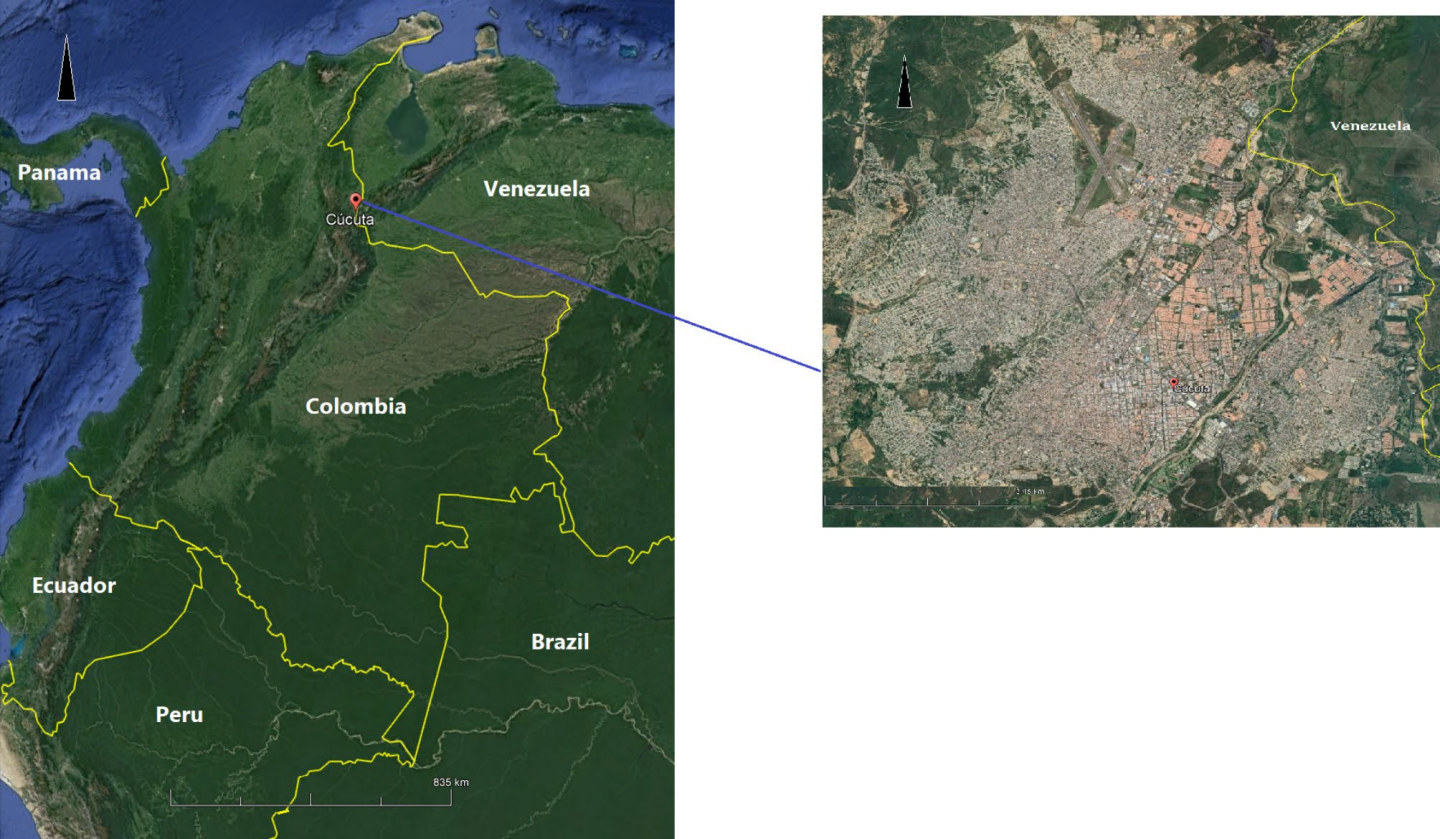
Geographical location and satellite view of Cúcuta (inset)

Cúcuta is the capital city of the North Santander Department located in the northeast of Colombia, sharing with Venezuela one of the most important international borders in Latin America in terms of social mobility and commerce [29] (Fig. 1). The city is generally flat with an average altitude of 320 m above the sea level, and an area of 1.176 km² which represents 5.65% of the Department area. It has an average temperature of 28 °C and average annual rainfall of 1,041 mm with small seasonal changes throughout the year. Cúcuta had an estimated population of 787,891, and in 2020 it was estimated that approximately 12% of the population were Venezuelan migrants [30]. The city’s geographic areas are categorised into 460 Urban Sections (USEC) established by the National Department of Statistics -DANE with an average size of 113,558 m^2^ (quartile-1: 83,216 m^2^; quartile-3: 175,929 m^2^) [31].

### Data

Data on the number of new consultations with a diagnosis of ARI from each health service in Cúcuta for the study period were obtained from the Public Health Department. As these data did not include geographical identifiers (i.e., place of residence), the number of ARI consultations per USEC was calculated using a method for spatial estimation of disease rates described elsewhere [18]. In brief, the consultations per USEC were estimated by categorising each health service into their corresponding USEC. Spatial zones were established for each health service considering the spatial extent of areas receiving their services and their level of complexity (i.e., primary, intermediate and high-complexity health services). A weight value was assigned to each spatial zone according to the proportion of the population in each USEC. The ARI consultations per zone were multiplied by the weight value to obtain the ARI cases per USEC. The rate of ARI (ARI-r) per USEC was then calculated as the total ARI cases divided by the USEC population.

Cúcuta has health services exclusive to the Venezuelan migrants, which are considered a particularly vulnerable population with higher risk of infectious diseases [32]. Data from these health services were included in the ARI-r calculations described above. In addition, an estimate of the ARI in the Venezuelan migrant population was calculated as the proportional rate of ARI in Venezuelan migrant health services per total USEC population, using the same method as the ARI-r. The total USEC population was used since no estimate of the Venezuelan migrant population in each USEC was available, noting there is high mobility in this population.

Data on air quality for Cúcuta are provided by the Institute of Hydrology, Meteorology and Environmental Studies -IDEAM, from monitoring stations strategically located which use the National Air Quality Monitoring Protocol [33]. These data include daily measures of particulate matter (PM_10_) (other pollutants are not measured by all the stations), therefore PM_10_ levels were obtained and averaged for the year 2018. Since there are only 3 monitoring stations, to calculate the PM10 value in any unmeasured location of the city, an inverse distance weighting interpolation model was implemented in ArcMap (v.10.6). Then the average PM10 level was calculated (as quintiles) in each USEC.

The age profile of each USEC were summarised by the percentages of the population younger than 15 years and the population older than 65 years, according to the 2018 census data. Other sociodemographic data included the Index of Multidimensional Poverty (IMP) that incorporates 15 indicators to estimate five dimensions of poverty, with higher values indicating poorer areas, and the Index of Population Economically Active (PEA) [34]. Climatic factors such as temperature and rainfall were not considered in this analysis as there is insufficient variance across the study area.

### Analysis

A Bayesian hierarchical spatial model was used to estimate the association of the ARI-r with the sociodemographic and environmental covariates in the study period. For the $i$-th USEC, the ARI consultations (${\eta }_{i})$ were modelled as

$${y_i} \sim Poisson\left( {{\lambda _i}} \right)$$

with the linear predictor defined on the logarithmic scale:

$${\eta }_{i}=\text{log}\left({\lambda }_{i}\right)=\alpha +{\beta }_{x}{X}_{xi}+\text{log}\left({pop}_{i}\right)+ {u}_{i}+ {st}_{i}$$

where $\alpha$ is the intercept; $X$ represents the vector of covariates (PM_10_, PEA, IMP, proportional ARI rate in migrants, population < 15 years and population > 65 years) with their respective regression coefficients ${\beta }_{x}$; and $\text{log}\left({pop}_{i}\right)$is the log of the population included as the offset. The parameters ${u}_{i}$ and ${st}_{i}$ are random effects representing the unstructured (non-spatial) and spatially structured residuals in the model, according to the Besag-York-Mollie (BYM) specification [35]. The BYM is a standard model for estimation of associations when the output variable is broken down into a random Poisson component, a spatially structured area (random effect component), and an unstructured random component, across the spatial units [36]. With this specification, ${st}_{i}$ is modelled using an intrinsic conditional autoregressive structure${w}_{i}$

$${w}_{i}=\frac{\sum _{j\in \mathcal{N}\left(i\right)}{ st}_{i}}{\#\mathcal{N}\left(i\right)} and {s}_{i}^{2}=\frac{{\delta }_{\upsilon }^{2}}{\#\mathcal{N}\left(i\right)}$$

which incorporates an adjacency matrix, where $\#\mathcal{N}$ is the number of USEC that share boundaries with the ${i}_{th}$ USEC.

The model has two hierarchies given by the parameters of the prior distribution (i.e. hyper-parameters) and the distribution of hyper-parameters, for which the prior of the structured and unstructured components need to be specified. To set these, models using four non-informative priors were compared before the analysis, using the Deviance Information Criterion (DIC) to establish the prior that produced the best fit model (i.e., lowest DIC). In addition, as there are no previous studies assessing the effect of different adjacency matrices of the Cúcuta’s USEC, five adjacency matrices were compared to identify the best fit model, following the method introduced by Cortes-Ramirez, Vilcins [37]. These preliminary analyses identified a prior ${\rm{log}}{\tau _v} \sim \log Gamma\left( {0.1,0.1} \right)$, $\log {\tau _\upsilon } \sim \log Gamma\left( {0.001,0.001} \right)$, and an adjacency matrix with a queen specification (i.e., including all neighbour USECs sharing a border per USEC) to produce the best fit in all models compared (details in the supplementary material). The queen specification was also used to test for spatial autocorrelation (i.e., the degree of the spatial association of each variable between neighbour area) [38] using the Moran test in R [39]. The ARI-r and all the predictors had a positive and significant Moran’s I estimate which confirmed the occurrence of spatial dependency in the data (details in the supplementary material).

The variance explained by the structured spatial component of the Bayesian model was obtained from a comparison between the posterior marginal variance of the structured and unstructured effects [27]. All Bayesian models were done in R using the R-INLA package that uses the Integrated Nested Laplace Approximation (INLA) [40] which is a standard alternative to Markov Chain Monte Carlo methods to produce regression estimates in analyses of spatially auto correlated data [25].

The occurrence of multicollinearity between the predictors was assessed as this can affect the interpretation of the regression coefficients. A Variance Inflation Factor (VIF) test was implemented where a $VIF \ge 5$ was considered collinearity [41], with no collinearity found for any of the predictors.

### Preliminary sensitivity analysis

To assess the independent effect of the predictors, the model with all covariates (full model) was compared with models that gradually eliminated variables without statistical credibility (credible intervals crossing the null value 1). To control for restricted selection of potential predictors and unstable covariates selection in this backward approach, the base model was compared with models that incorporated all combinations of adding one or more of the other covariates without statistical credibility. This process produced models with similar results to the model obtained by backwards elimination in terms of the statistical credibility of the predictors.

### Specific risk of ARI per USEC and cluster analysis

The distribution of the USEC-specific relative risk of ARI (compared to the whole of Cúcuta) was mapped using the marginal random effects in the full model. Since the probability of increased risk calculated in spatial analyses can be used as a decision rule threshold [42], the probability of a 1.5-fold higher risk of ARI (i.e. excess risk > 1.5) was calculated from the posterior distribution. For the identification of hotspots of risk, the USEC with increased risk, irrespective of magnitude, that were surrounded by USEC also having increased risk were labelled high-high risk USEC or hotspots of risk, following similar approaches to identify clusters of risk in other spatial epidemiological analyses [43]. The same approach was used to identify cold spots (low-low risk USEC). All maps were produced with the R-package T-map [44].

## Results

There were 118,469 cases of ARI in Cúcuta over the study period with an average ARI-r of 25.8 cases per 100 people. Table 1 shows the summary statistics of the ARI-r and the predictors.

**Table 1:** Summary of the acute respiratory infections rate and socio-environmental variables in Cucuta, 2018

Variable	Mean	St. Dev.	Min	Q1	Q3	Max
ARI rate*	25.77	22.63	14.29	19.04	25.03	372.86
Proportional ARI rate - migrants	3.24	2.60	0.00	1.56	4.04	25.71
Average PM_10_ (µg/m^3^)	45.00	2.90	39.13	42.60	47.42	49.58
Index of Population Economically Active (PEA)	51.53	3.57	35.42	49.40	53.30	70.49
Index Multidimensional Poverty (IMP)	23.64	15.32	0.24	12.43	31.91	85.62
Population < 15 years (%)	25.52	23.04	10.00	18.65	26.49	29.71
Population > 65 years (%)	48.86	43.16	0.00	21.97	59.54	68.97

Notes. ARI: Acute Respiratory Infections; Q1 and Q3: first and third quartile respectively. *Per 100 people

**Fig. 2:**
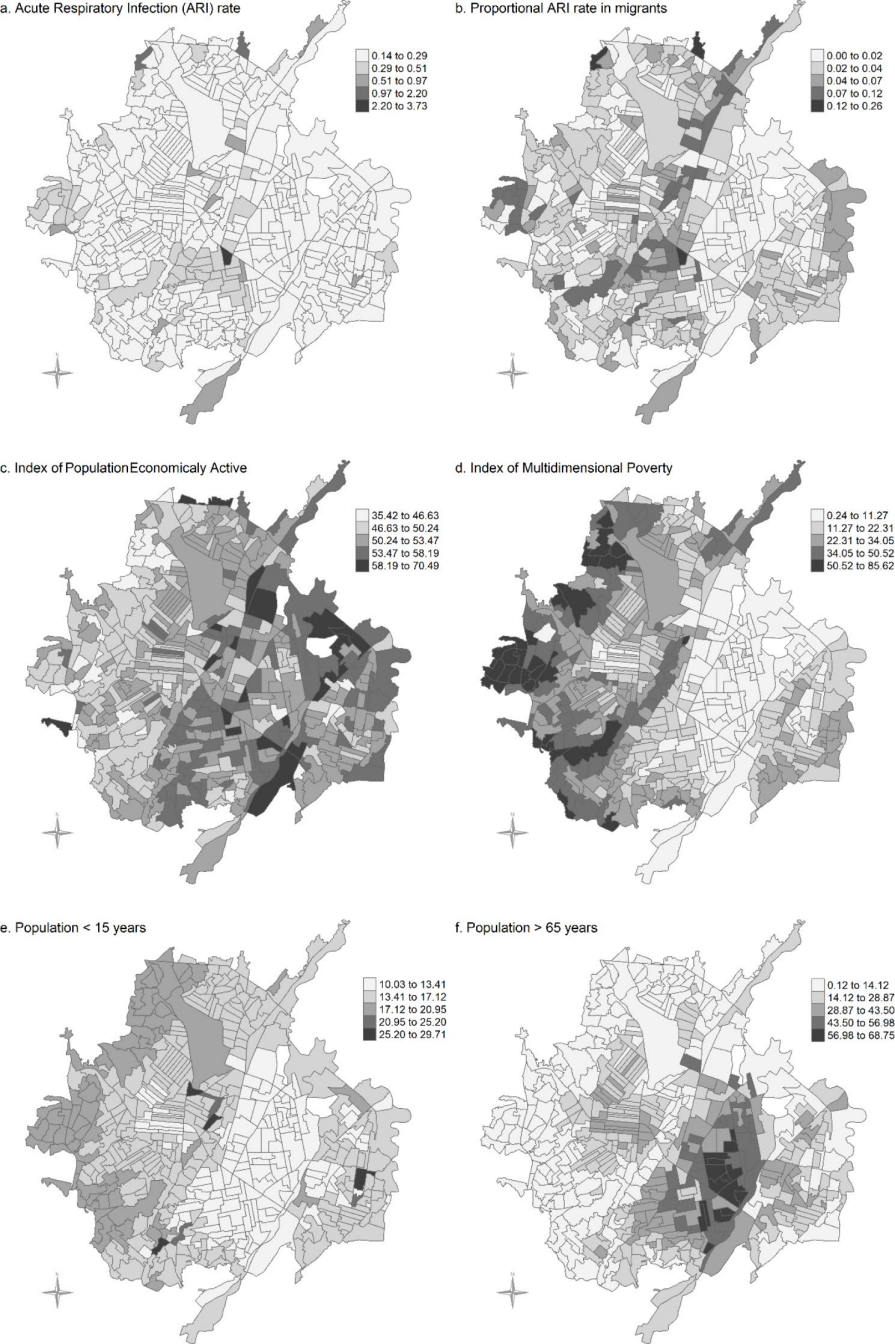
Distribution of the ARI rate and sociodemographic predictors in the 460 Cúcuta’s Urban Sections. The population < 15 years and population > 65 year are expressed per 100 people

The spatial distribution of the ARI-r and the predictors is shown in Fig. 2.

The backward elimination modelling approach identified only three covariates with coefficients with statistical credibility: the proportional ARI rate in migrants, the IMP and the proportion of population < 15 years. The model with only these three variables did not have a better fit than the full model as the DIC difference was < 2 —i.e., it does not account for a better model fit [45]. Table 2 shows the exponentiated posterior mean for the fixed effects (regression coefficients) of the full model. A positive association was found for the proportional ARI rate in migrants, the population < 15 years and the IMP with the ARI-r. Both hyperparameters were associated with an increased ARI-r risk. The exponentiated regression coefficients can be interpreted as relative risks (i.e., an increase of 1 unit in the proportional ARI rate in migrants, the population < 15 years and the IMP is associated with a 1.25-, 1.08- and 1.04 increase of the ARI-r in Cúcuta, respectively). The proportion of population > 65 years had a positive effect on the ARI-r although the CI crossed the null value. The positive association of both hyperparameters indicates the impact of the incorporation of the BYM (spatial) specification in the model. Some of the variability was explained by the spatial structure of the USEC incorporated in the model (proportion of spatial variance = 1.8%).

**Table 2:** Exponentiated posterior mean with 95% credible intervals (CI) of the spatial regression model

Predictor	Exp (Posterior mean)	95% CI
ARI rate -migrants	1.25	**1.22–1.27**
Average PM_10_	0.97	0.91–1.03
Index of Population Economically Active	0.99	0.97–1.01
Percentage population < 15 years	1.08	**1.06–1.1**
Percentage population > 65 years	1.01	0.97–1.04
Index Multidimensional Poverty	1.04	**1.01–1.08**
**Hyperparameters**:		
Precision for unstructured component	62.16	**42.48–86.57**
Precision for structured component (spatial)	18.51	**11.7–28.51**
Variance for the spatial structured effect	0.018	
Deviance Information Criterion (DIC)	4245.51	

**Fig. 3:**
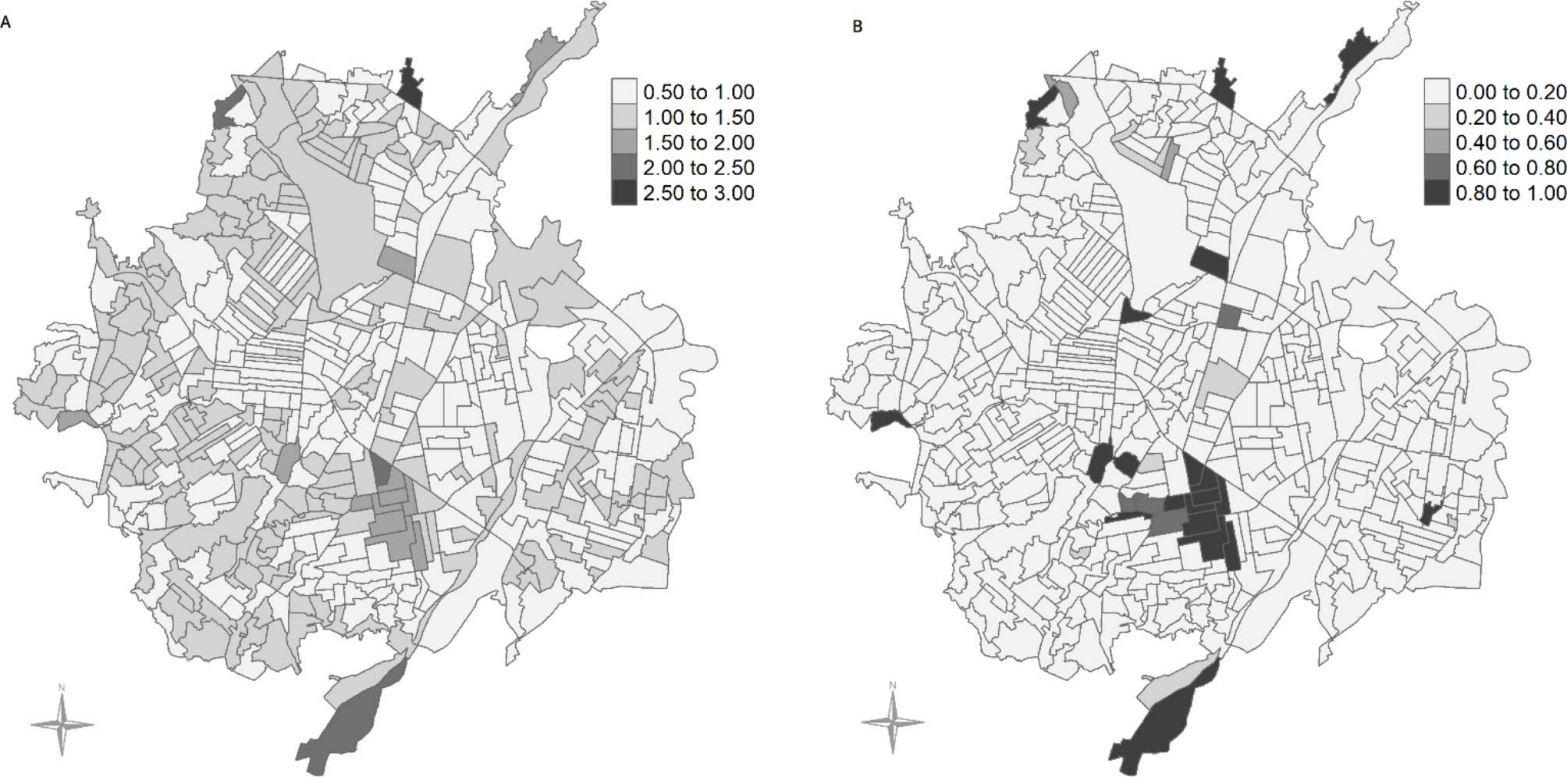
**A**. Distribution of the Urban Section (USEC) specific relative risk of ARI | **B**. USEC-specific probability of risk greater than 1.5

Figure 3 (A) shows the relative risk of ARI in each USEC (compared to the whole of Cúcuta) once sociodemographic and environmental factors are taken into account in the full model. Sixteen USEC had a 1.5 or higher relative risk of ARI, of which 4 had a relative risk greater than 2-fold in the north, central and south regions of Cúcuta. Multiple USEC with a risk > 1.5 appeared clustered in the central region. Figure 3 (B) shows the distribution of the probability of risk to be greater than 1.5 (excess of risk > 1.5). All 16 USEC with a 1.5-fold relative risk had an 80% or more probability of increased risk.

**Fig. 4:**
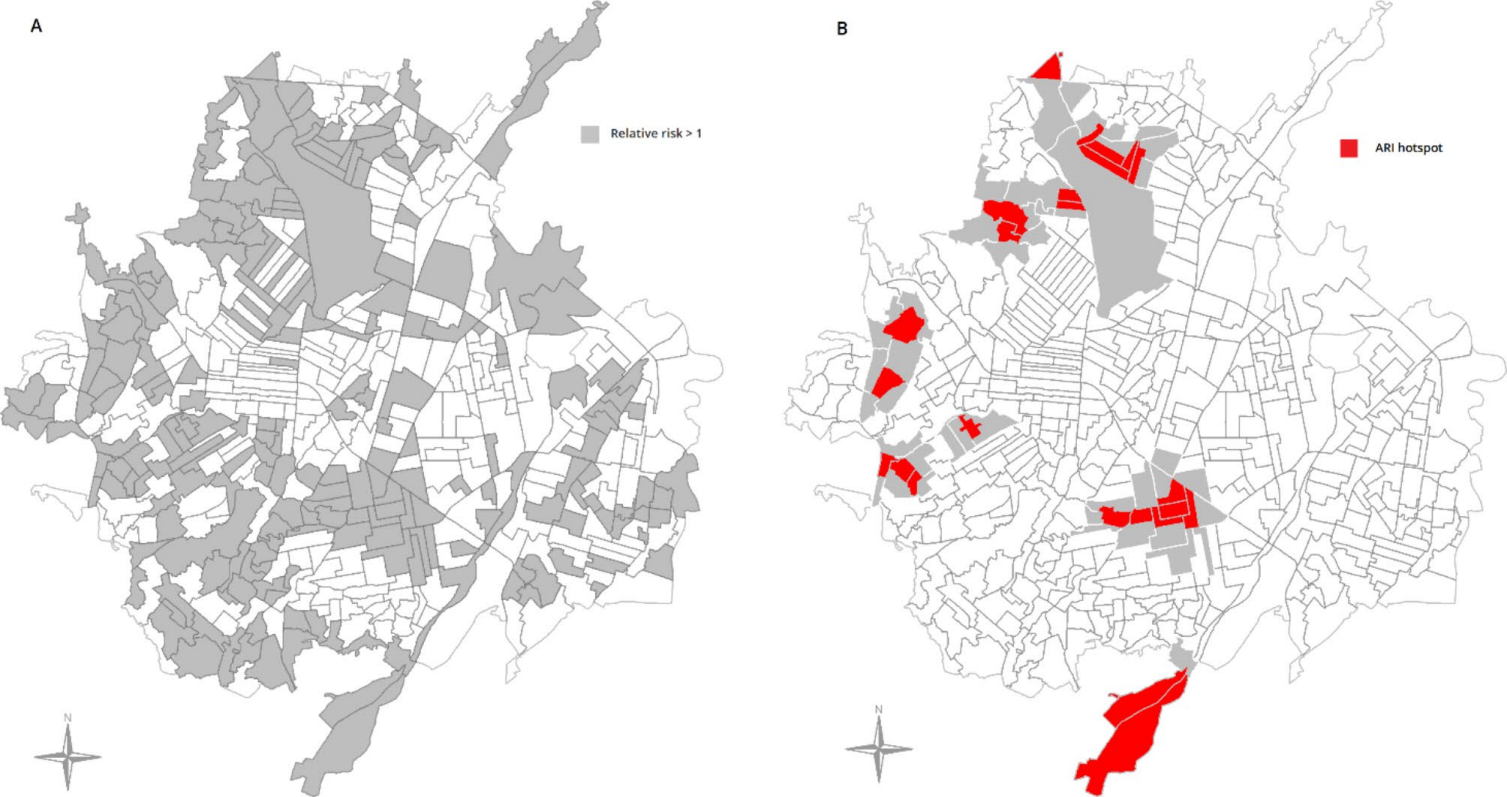
**A**. Distribution of USEC with higher risk, irrespective of magnitude (relative risk of ARI > 1). | **B**. Hotspots of risk (in red) —i.e., high-risk USEC (in red) surrounded by USEC with also high-risk (in grey)

Figure 4 (A) shows the cluster analysis of USEC with higher relative risk. There were 217 USEC with a higher risk of ARI compared with the whole of Cúcuta. Of these, 24 USEC surrounded by USEC with higher risk (high-high risk USEC, or hotspots) were identified (Fig. 4B). More than one hotspot clustered together in central, north, west and south regions while some isolated hotspots were found in north and west regions. There were no low-low risk USEC identified. Several of the hotspots occurred on the northern and southern edges of the city. As only data from Cúcuta’s USEC was included, it should be noted that the number of neighbours for each USEC on the city perimeter is artificially reduced, which results in a less restrictive specification when determining hotspots compared with USEC far from the city edges.

## Discussion

In this study we calculated and mapped the predicted risk of ARI by USEC in Cúcuta, Colombia, after adjusting for the effect of socioenvironmental risk factors. The regions with higher risks were found in central, south, north and western Cúcuta. The most important factors associated with the risk of ARI in the whole of the city were the rate of ARI in migrants, the percentage of population < 15 years and the Index of Multidimensional Poverty. The analysis implemented a Bayesian hierarchical model that allowed further identification of the USEC with higher probability of increased risk and the hotspots risk. To the best of our knowledge, this is the first study to map the risk of ARI in census districts in a capital city in Colombia.

The analysis shows an increased risk of ARI associated with two well-established factors as important determinants of respiratory infections in Colombia and globally: younger age and poverty. Previous research has found a mortality proportion due to ARI in people under 19 years up to 27% in Colombia with the North Santander region having a comparative higher mortality [46], while children under five years have the highest incidence and DALY rates of upper respiratory tract infections globally [47]. Higher socioeconomic inequality has been associated with an increased risk of ARI, according to the global burden of disease study 2019 [48], which concurs with previous studies showing higher ARI mortality rates in poor Colombian municipalities nationally and in large cities such as Bogota and Manizales [49, 50]. These prior analyses have been undertaken at the national or regional level without regard to the risk variation within big urban settings such as Cúcuta. The current study also identifies these risk factors, but at a much smaller spatial scale. These results demonstrate how the patterns of risk of ARI in a city with significant international mobility in Latin America can be determined, and would support the understanding of the variation in the risk of ARI at a suburban level in large cities in Colombia and the region.

Another factor associated with the risk of ARI in Cúcuta was the proportional rate of ARI in migrants from Venezuela. While this association is expected (as it is proportional to the ARI rate), we could verify the important effect that the ARI rate in this particular population has on the overall risk of ARI, considering its spatial distribution. These findings support previous estimations of increasing risk of infectious diseases in Venezuelan migrants in Colombia and other countries in the vicinity including Brazil and Peru [32, 51, 52]. This is an especially vulnerable population as the political and economic instability in Venezuela in the last 10 years has created increasing and sometimes massive migrations to Latin American countries, mostly to Colombia and its departments close to the international border [53]. These conditions have exposed the migrant population to unfavourable environmental and or social conditions that facilitate the emergence of infectious diseases [54]. Similar trends of increased morbidity in immigrants in other regions such as North America have been observed, mostly associated with unequal poverty levels or reduced access to appropriate health care [10]. The increased morbidity in Venezuelan immigrants increases the demand of health services, reinforcing the need for more health care providers for this group or better access to public health facilities. These findings can support the identification of specific areas and or communities in Cúcuta for targeted public health strategies.

The Bayesian spatial approach of the analysis allowed the identification of substantial geographic variation in the risk of ARI within Cúcuta, with 16 USEC estimated to have a 1.5 or higher risk. Although somewhat clustered, these USEC were dispersed throughout the city rather than in one specific region and were generally surrounded by other areas of heightened risk. This is consistent with spatial analyses in other cities such as Edmonton, Canada [10] and Ho Chi Minh City in Vietnam [7] that identified spatial heterogeneity in the risk of ARI at the suburban level. Our findings support the importance of studying the spatial heterogeneity of the risk of infections in epidemiological analyses to increase the accuracy of prediction of outbreaks and to identify potential underlying factors determinant of infectious diseases [13]. The analysis also identified the USEC with 80% or more probability of a 1.5-fold risk. These estimates can be of great value in public health decision-making as these can be used as rules to establish priorities in health strategies and or interventions, an approach already used in other contexts [55, 56]. Although we used ARI records of 2018 only due to data limitations of the health department, the risk of these diseases identified in this study is an important reference for future analyses, especially considering the potential rebound of respiratory infections after the relieve in the control measures imposed during the Covid-19 period [57]. The results have implications for both targeted prevention measures and planning for adequate care facilities in Cúcuta, while the method used in this study can be applied in other urban contexts in Colombia and globally.

There are important limitations in this study, especially regarding the analysis of data of ARI at an aggregate level (per USEC) and the subsequent risk of ecological bias (i.e., analysis of data at the USEC level can produce spurious associations). To address this issue, the analysis used a BYM model which incorporates random effects on the USEC, in addition to the adjustment for socioenvironmental covariates. Although the use of both these approaches is deemed to help reduce the risk of ecological bias [58, 59], further analyses at the individual level are required to provide supplementary evidence on the causality of ARI in the city. In addition, the spatial estimation method to calculate the cases of ARI per USEC makes assumptions about the populations accessing health services across the city [18] which might not produce an accurate count of cases and increase the risk of dispersion of the ARI rates. We used a multiple regression with a spatial component in a Bayesian hierarchical model that can reduce the impact of overdispersion. However, further research that incorporates specific residential location data and risk factors at the individual level are needed to identify patterns of causality.

Another limitation of the ARI data was the lack of individual characteristics such as age and gender, meaning that the rates could not be sex-adjusted or age-adjusted. While this can affect the comparability of the ARI rate between the USEC due to underlying differences in the age and gender structure of their population, the study aimed to identify areas with increased risk of ARI for targeting health resources, therefore regardless of this, the areas with higher proportions of young people and increased ARI risk would need more public health support. Nevertheless we used a spatial model that reduces the potential effect of differences in the age structure because it smooths the estimates across the USEC and mitigates the impact of outliers [27]. Standardisation of rates by age and or sex should be considered in future analyses using morbidity data on ARI in Cúcuta, looking at the causes of ARI other than age such as poverty or living conditions to remove the increased risk associated with age and identify areas with unexplained risk of ARI.

## Conclusions

A spatial statistical approach can be used to explain the distribution of the risk of respiratory infections in suburban areas of large cities such as Cúcuta, Colombia. The Bayesian spatial model used identified areas with higher risk of morbidity due to these diseases in central, south, north and west of the city, and 24 specific urban sections identified as statistical hotspots of risk. The most relevant risk factors for these diseases were the proportion of population younger than 15 years, the index of multidimensional poverty and the high rate of respiratory infections in migrants from a neighbouring country. The methods used in this study can be implemented in disease mapping analysis in other regions in Colombia and globally to identify communities at risk and support decision making in the public and private health sectors. Whereas this analysis estimated and mapped the risk for small geographical areas, further research with individual-level data is needed to address the causality of respiratory infections in Cúcuta.

## Electronic supplementary material

Below is the link to the electronic supplementary material.

Supplementary Material 1

## Data Availability

All data produced in this study are presented in the results or the supplementary material except for the raw data on acute respiratory infections to be accessed only for the analysis, according to the ethics approval conditions. Any data request should be sent to the corresponding author.
