# Supplementary material for: Mapping the risk of respiratory infections using suburban district areas in a large city in Colombia
Source: BMC Public Health. 2023 Jul 20;23:1400. doi: 10.1186/s12889-023-16179-5 (PMC10360249; doi:10.1186/s12889-023-16179-5)
Supplement: Supplementary file 1 — Supplementary Material 1 [file 12889_2023_16179_MOESM1_ESM.docx]

Mapping the risk of respiratory infections using suburban district areas in a large city in Colombia

Javier Cortes-Ramirez ^1,2,3^; Michelle Gatton ^4^; Juan D. Wilches-Vega ^3^; Helen J. Mayfield ^5^; Ning Wang ^6^; Olga M. Paris-Pineda ^3^; Peter D. Sly ^2^.

1. Centre for Data Science. Queensland University of Technology
2. Children’s Health and Environment Program, Child Health Research Centre. The University of Queensland
3. Faculty of Health. University of Santander
4. Centre for Immunology and Infection Control. Queensland University of Technology
5. School of Public Health. The University of Queensland
6. National Centre for Chronic and Noncommunicable Disease Control and Prevention. Chinese Centre for Disease Control and Prevention

Corresponding author: Dr Javier Cortes-Ramirez. Address: O Block D Wing Room D722. Ring Road, Kelvin Grove Campus. Queensland University of Technology, Victoria Park Road. Kelvin Grove QLD 4059 Australia. E: [javier.cortesramirez@qut.edu.au](mailto:javier.cortesramirez@qut.edu.au)

**Supplementary material**

**Preliminary analysis. Comparison of priors and adjacency matrices**

The following priors were used to compare the better of fit of the Bayesian spatial models:

$$Prior 1. \log\tau_{v}\sim\log Gamma (0.1, 0.01), \log\tau_{\upsilon}\sim\log Gamma (0.1, 0.01)$$

$$Prior 2. \log\tau_{v}\sim\log Gamma \left( 0.1, 0.1 \right), \log\tau_{\upsilon}\sim\log Gamma \left( 0.001, 0.001 \right)$$

$$Prior 3. \log\tau_{v}\sim\log Gamma \left( 0.5, 0.001 \right), \log\tau_{\upsilon}\sim\log Gamma \left( 0.5, 0.001 \right)$$

$$Prior 4. \log\tau_{v}\sim\log Gamma (1, 0.005), \log\tau_{\upsilon}\sim\log Gamma (1, 0.005)$$

The use of five adjacency matrices (AM) in the spatial specification of the model was compared to identify the best fit using the DIC. These AM included a queen-specification (i.e., all surrounding neighbours as in a chess game) (58), and four adjacency matrices set using a K nearest neighbour (KNN) algorithm. The KNN sets a “K” number of neighbours assigned to each geographical area (59). The K values 5, 7, 9, and 11 were included, considering the guidelines introduced by (58), Hassanat, Abbadi (60). The coordinates of each USEC centroid were calculated in ArcMap to estimate the distance between neighbours. Table S2 shows the DIC of models using each of the priors and the adjacency matrices.

**Table S1.** Deviance Information Criterion of the models compared

| Adjacency matrix specification | Prior 1 | Prior 2 | Prior 3 | Prior 4 |
| --- | --- | --- | --- | --- |
| Queen | 4264.46 | 4262.83 | 4264.86 | 4265.10 |
| KNN=5 | 4303.87 | 4304.58 | 4304.09 | 4303.93 |
| KNN=7 | 4303.94 | 4303.88 | 4303.64 | 4303.62 |
| KNN=9 | 4298.64 | 4298.14 | 4300.70 | 4298.64 |
| KNN=11 | 4293.10 | 4299.14 | 4296.08 | 4295.54 |

*Notes. KNN:* *K nearest neighbour*
